# Panel discussion: early interventions after traumatic events

**DOI:** 10.3402/ejpt.v6.28636

**Published:** 2015-06-11

**Authors:** Filip K. Arnberg, Kristina Bondjers, Josefin Sveen

**Affiliations:** National Centre for Disaster Psychiatry, Department of Neuroscience, Uppsala University, Uppsala, Sweden

Early interventions for psychological consequences of traumatic events should be based on the best available evidence while they are delivered in a great variety of contexts (Litz, 2015). Additionally, these interventions are to be delivered in collaboration with local organizations and a wide range of other first responders, including other emergency healthcare services as well as journalists and media (Newman & Drevo, 2015). In this complex setting, it can be challenging for psychosocial services to identify and utilize applicable research findings while supporting idiographic recovery processes.

The panelists discussed diverse aspects related to evidence and practice of early interventions. Biological aspects include developing research on prevention by means of pharmacologically reducing hyperarousal and increasing the likelihood of pro-social behavior. The evidence, benefits, and drawbacks of very early psychological structured interventions were highlighted, including the use of new technology as stand-alone interventions or supplementary modules (Olff, 2015; Olff, Van Zuiden, & Bakker, 2015). The panelists also discussed the need for monitoring what interventions are given, and to whom, in order to evaluate their effects in naturalistic settings.

The national societies for psychotraumatology may serve as a link to local and regional initiatives for improving psychosocial interventions to international societies. For example, they can provide nationally adapted information about developments in science and practice, and, importantly, facilitate communication and interaction between local researchers and clinicians across countries.

This conference was funded by a grant from The Swedish Foundation for Humanities and Social Sciences (F14-1747:1).
